# Metabolic Drug Response Phenotyping in Colorectal Cancer Organoids by LC-QTOF-MS

**DOI:** 10.3390/metabo10120494

**Published:** 2020-12-01

**Authors:** Sylvia K. Neef, Nicole Janssen, Stefan Winter, Svenja K. Wallisch, Ute Hofmann, Marc H. Dahlke, Matthias Schwab, Thomas E. Mürdter, Mathias Haag

**Affiliations:** 1 Dr. Margarete Fischer-Bosch-Institute of Clinical Pharmacology, Stuttgart, Germany and University of Tuebingen, 70376 Tuebingen, Germany; Sylvia.Neef@ikp-stuttgart.de (S.K.N.); Nicole.Janssen@ikp-stuttgart.de (N.J.); Stefan.Winter@ikp-stuttgart.de (S.W.); Svenja.Wallisch@ikp-stuttgart.de (S.K.W.); Ute.Hofmann@ikp-stuttgart.de (U.H.); Marc.Dahlke@rbk.de (M.H.D.); Matthias.Schwab@ikp-stuttgart.de (M.S.); Thomas.Muerdter@ikp-stuttgart.de (T.E.M.); 2 Department of Surgery, Robert-Bosch Hospital, 70376 Stuttgart, Germany; 3 Departments of Clinical Pharmacology, and of Pharmacy and Biochemistry, University of Tuebingen, 72074 Tuebingen, Germany; 4 Cluster of Excellence iFIT (EXC 2180), Image-Guided and Functionally Instructed Tumor Therapies, University of Tuebingen, 72074 Tuebingen, Germany

**Keywords:** metabolomics, lipidomics, metabolic profiling, organoids, colorectal cancer, QTOF, LC-MS

## Abstract

As metabolic rewiring is crucial for cancer cell proliferation, metabolic phenotyping of patient-derived organoids is desirable to identify drug-induced changes and trace metabolic vulnerabilities of tumor subtypes. We established a novel protocol for metabolomic and lipidomic profiling of colorectal cancer organoids by liquid chromatography quadrupole time-of-flight mass spectrometry (LC-QTOF-MS) facing the challenge of capturing metabolic information from a minimal sample amount (<500 cells/injection) in the presence of an extracellular matrix (ECM). The best procedure of the tested protocols included ultrasonic metabolite extraction with acetonitrile/methanol/water (2:2:1, *v/v/v*) without ECM removal. To eliminate ECM-derived background signals, we implemented a data filtering procedure based on the *p*-value and fold change cut-offs, which retained features with signal intensities >120% compared to matrix-derived signals present in blank samples. As a proof-of-concept, the method was applied to examine the early metabolic response of colorectal cancer organoids to 5-fluorouracil treatment. Statistical analysis revealed dose-dependent changes in the metabolic profiles of treated organoids including elevated levels of 2′-deoxyuridine, 2′-*O*-methylcytidine, inosine and 1-methyladenosine and depletion of 2′-deoxyadenosine and specific phospholipids. In accordance with the mechanism of action of 5-fluorouracil, changed metabolites are mainly involved in purine and pyrimidine metabolism. The novel protocol provides a first basis for the assessment of metabolic drug response phenotypes in 3D organoid models.

## 1. Introduction

Around one decade ago the groups of Hans Clevers [1] and Yoshiki Sasai [2] revolutionized cell culture with their pioneering work in the field of organoids. Organoids are stem cell-derived 3D structures that mimic the in vivo situation more precisely in terms of architecture, cell-type composition and self-renewal properties compared to current 2D cell culture models [1,2,3,4,5]. Thus, organoid cultures have emerged as a promising model in the fields of drug discovery, personalized medicine and cancer research [6,7]. In particular, in the context of cancer, organoids gained great importance as they can be generated from patient biopsies allowing the analysis of tumor evolution, heterogeneity and even patient-specific treatment responses [8]. With colorectal cancer (CRC) being the third most common cancer in both sexes [9] and an overall response rate of 17–36% to standard chemotherapy [10], it is highly relevant to identify biomarkers that accurately predict the patient response.

Like other cancer entities [11,12] CRC undergoes specific metabolic reprogramming during carcinogenesis [13] including dysregulation of energy [13] and lipid metabolism [14,15]. Therefore, metabolism has been suggested as a targetable vulnerability in CRC [16]. Further, a comprehensive analysis of metabolic changes upon treatment might help to identify a composite set of metabolites serving as a biomarker for the patient response. Consequently, the combination of well-established culture strategies for primary CRC organoids [1,4] and non-targeted metabolomic and lipidomic profiling [17,18] is a promising approach in drug research and biomarker discovery. The combination of these techniques enables a high-throughput drug screening using a patient derived model that mimics the in vivo situation more closely and may support new approaches towards personalized therapies.

In contrast to other omics-technologies including genomics [8], transcriptomics [19] and proteomics [20] metabolomics is rarely used for characterization of organoid models. Whereas protocols for cell culture metabolomics are well established [21], only a few studies captured the metabolome from organoids by using NMR [22] and targeted [23] or non-targeted [24,25,26] LC-MS based profiling. In terms of non-targeted metabolomic profiling, there is an acute lack of optimization studies addressing problems such as the required sample amount and sampling conditions. Moreover, the influence of background signals derived from the protein based hydrogel, which is often indispensable for organoid culturing as a basal membrane matrix, on metabolomics data preprocessing has not been addressed comprehensively.

In this work, we describe the evaluation of an optimized extraction protocol enabling untargeted metabolomic and lipidomic profiling of CRC organoids grown in the extracellular matrix (ECM) via hydrophilic interaction liquid chromatography (HILIC)- and reversed phase liquid chromatography (RPLC)-QTOF-MS [17].

## 2. Results and Discussion

### 2.1. Assessment of Sample Preparation for Metabolomic and Lipidomic Profiling in CRC Organoids

**Figure 1:**
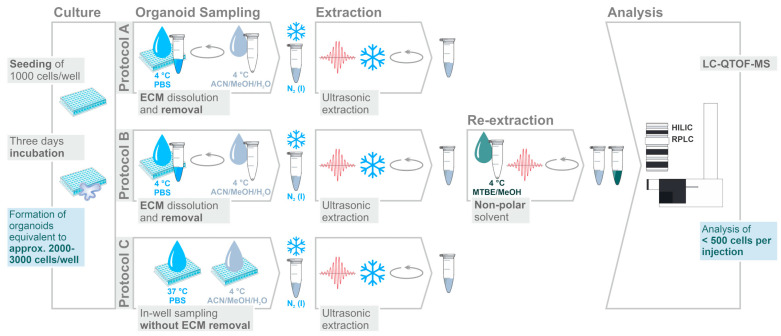
Extraction protocols evaluated for metabolomic and lipidomic profiling of colorectal cancer (CRC) organoids using LC-QTOF-MS after dual LC separation by HILIC and RPLC. The number of seeded cells/well was determined using an hemocytometer. Cell numbers after incubation were approximated based on the doubling time (3.4 days) as determined in concomitant experiments. Cell numbers for LC-MS analysis were calculated based on the solvent volumes used for sample preparation (optimized protocol C, see Supplementary Figure S7). Resulting extracts were dried and reconstituted in appropriate solvent prior to LC-QTOF-MS analysis.

To maintain their 3D-structure, organoids need to be cultured surrounded by an ECM. The ECM used in our experiments is a gelatinous protein mixture that is liquid at low temperatures but polymerizes upon incubation at 37 °C. In order to establish a sample preparation protocol for non-targeted metabolomic and lipidomic profiling of CRC organoids cultured in ECM, we tested different organoid sampling procedures (see Figure 1). Washing with phosphate-buffered saline (PBS) was carried out at two different temperatures. First, washing with 4 °C PBS (protocols A and B) was chosen, as cold washing is a commonly used procedure for metabolism quenching and to remove extracellular medium components for untargeted metabolomics of cultured cells [27]. As ECM becomes depolymerized (i.e., liquefied) in the cold, washing at physiological temperature (37 °C) was tested as alternative (protocol C). The higher temperature keeps matrix proteins in the polymerized state and in consequence retains organoid cells embedded in their matrix.

For all three protocols, metabolite recovery from organoids was achieved by extraction with the solvent mixture acetonitrile/methanol/water (ACN/MeOH/H_2_O, 2:2:1, *v/v/v*), which has previously been applied to targeted [28] and untargeted [18] metabolic profiling of human cells and organoids [24] in a slightly modified composition (3:5:2, *v/v/v*). However, the rather polar nature of the solvent may compromise the recovery of non-polar lipids. As two-step extraction protocols are frequently applied to increase metabolite coverage [17,29], a potential benefit of organic re-extraction with monophasic methyl tert-butyl ether/methanol (MTBE/MeOH, 3:1, *v/v*), as part of the protocol B, was investigated. Method quality rating was achieved based on the number of metabolites that could be detected (*p*-value <0.05, Welch’s test, fold change [FC] > 1, *n* = 5 technical replicates) above ECM blank samples. Further method repeatability was assessed by the median coefficients of variation (CVs) of those metabolites.

In total, 107 unique metabolites could be detected (above ECM blank, Supplementary Figures S1–S4) with an overlap between protocols ranging from 12% to 60% depending on the LC-MS mode (Supplementary Figure S5). As becomes evident from the diagrams the sample preparation protocol C resulted in the highest number of polar molecules (Table 1 and Supplementary Figures S1B, S2B, S5A and S5B) and lipids (Table 1 and Supplementary Figures S3C, S4C, S5C and S5D) compared to protocols A and B. Notably, the overlap between RPLC and HILIC was only 6% for protocol C (Supplementary Figure S6) indicating an increase in metabolome coverage by employing multiple LC-MS methods used together with this sample preparation protocol. In particular, the quantity of phospholipid species (e.g., belonging to phosphatidylcholines (PCs) and phosphatidylinositols (PIs)) and sphingolipids (e.g., ceramides (Cers) and sphingomyelins (SMs)) was markedly improved by “in-well sampling” without ECM removal (protocol C) compared to the ECM dissolution and removal procedure (protocols A and B). An explanation for the lower number of lipid species detected with protocols A and B may be due to the additional centrifugation step, which likely retains residual lipids in the supernatant. An alternative scenario may be metabolite leakage during ECM dissolution and removal, as indicated by reduced signal intensity of lipid-like species in colon carcinoma cells after cell washing with PBS or water [27]. Both scenarios however warrant further investigation. Notably, albeit protocol C resulted in a general improvement of the detection of phospholipids, only two-step extraction (protocol B) allowed for the analysis of non-polar triacylglycerols (e.g., TAG 52:2, Supplementary Figure S3B). Hence, as the formation of TAG-containing lipid droplets (LD) has been associated with tumorigenicity [30] in intestinal stem cells, sequential extraction may be an appropriate procedure to examine neutral lipid metabolism in CRC organoids. While protocol B enabled most repeatable measurements of lipids (median CV < 9%, Table 1), protocol C represented the best compromise between metabolite coverage (17–54 metabolites for all modes) and repeatability as indicated by median CVs 10–27% (Table 1). Thus, protocol C (37 °C PBS washing and “in well” sampling) offers a fast and simple procedure for repeatable metabolic phenotyping of colon cancer organoids with reasonable coverage of metabolites and lipids. In particular, the protocol enables rapid quenching of metabolic reactions in less than 1 min and metabolite extracts from 30 samples are ready for LC-MS analysis within less than 2 h. Such advantages of fast extraction with minimal cell manipulation are in accordance with recent findings from protocol optimization experiments for tumor spheroid metabolomics, where the optimized protocol consisted of rapid on plate washing followed by cold methanol extraction [31].

### 2.2. Filtering of ECM-Derived Background Features by Fold Change and p-Value

Like other high throughput assays, non-targeted metabolomic profiling experiments are subject to variations due to unwanted experimental or biological noise. Especially for 3D organoids, the basement membrane matrix, which is inherently composed of biomolecules (e.g., structural proteins), represents a rich source of signals that can affect downstream normalization and statistical analysis (i.e., reduced statistical power due to high number of tests). Thus, filtering of background features is an important step that has not yet received sufficient attention in the untargeted metabolomics analysis of cultured organoids.

**Figure 2:**
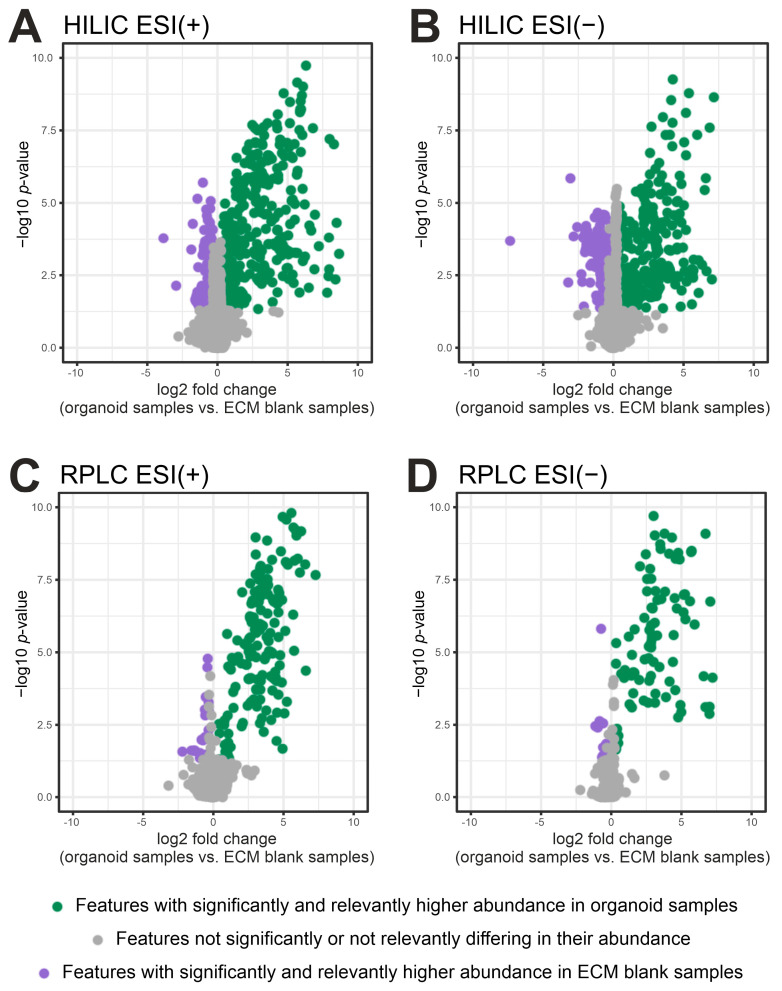
Volcano plots comparing the abundance of features detected in organoid samples (*n* = 5) and ECM-blank samples (*n* = 3): (**A**) HILIC ESI (+) mode; (**B**) HILIC ESI (−) mode; (**C**) RPLC ESI (+) mode; (**D**) RPLC ESI (−) mode. Features with significantly and relevantly higher abundance in organoid samples (fold change (FC) > 1.2, *p* < 0.05, HILIC ESI (+)/(−): 311/299 features and RPLC ESI (+)/(−): 149/92 features) are colored in green and are considered to be cell derived. Features with significantly and relevantly higher abundance in ECM-blank samples (FC < 0.8, *p* < 0.05, HILIC ESI (+)/(−): 113/117 features and RPLC ESI (+)/(−): 25/13 features) are colored in purple. Grey dots represent features not significantly or not relevantly differing in their abundance (HILIC ESI (+)/(−): 1170/735 features and RPLC ESI (+)/(−): 406/217 features). Purple and grey features were considered to represent uninformative background signals and were removed prior to subsequent statistical analysis.

The use of fold change (FC) cutoffs (biological signal/blank signal) to remove features with insufficient abundance in biological samples is a common filtering method [32,33]. The two-step filtering procedure that we had chosen, which was based on a fold change (FC) of 1.2 (mean abundance of ECM blank samples + 20%) and an uncorrected significance level of 5% (i.e., Welch’s *t*-test *p*-value < 0.05, comparing biological samples vs. ECM blank samples), retained 19.5% and 26% of features in HILIC and 25.7% and 28.6% in RPLC in the positive and negative mode, respectively (Figure 2, green dots). The majority of features was filtered out (>70%, Figure 2, grey and purple dots) and was considered to be uninformative background derived from cell culture environment and other contaminants (e.g., vials or solvents) [34].

Such a proportion of eliminated features are typically achieved by other procedures that also make use of blank samples [35] where 74% and 76% of “low quality features” were excluded from publicly available urine and cell line test datasets, respectively. Notably, our two-step filtering procedure further removed features with high variability (max CV = 214% before and 76.1% after filtering, Supplementary Figure S8) hence demonstrating a beneficial effect of background noise elimination on the repeatability of organoid sample analysis.

We further observed that 268 features exhibited higher abundance in ECM blank samples compared to organoid containing samples (FC < 0.8, *p*-value < 0.05, purple dots in Figure 2). This observation could be attributed to matrix effects [36], to components present in culture medium and enriched in the ECM in the absence of cells [37] or to ECM derived components [36,38] that are taken up and metabolized in the presence of cells. CEU mass mediator batch search [39,40] based on their exact mass revealed that some of these compounds could be di- and tripeptides (nine features, see Supplementary Tables S6–S9) thus pointing to subproducts of proteins (i.e., laminin or collagen) as major Matrigel components [41]. In addition, phospholipid species (19 features), which were previously reported to be ECM derived contaminants [36], were reported by exact mass search.

Further, the search indicated that small molecules like organic acids and free fatty acids may contribute to the complex ECM composition. A full list of exact masses and potential annotation is provided in the supplementary material (see Supplementary Tables S6–S9). However, a detailed proteomic and metabolomic characterization of the used ECM is beyond the scope of our study and warrants further investigation. In this regard, the use of mass spectrometry-peptidomics will be pivotal to bridge the gap between proteomics and metabolomics [42] and to characterize the molecular composition of ECMs in much more detail.

Taken together, we introduced a simple two-step filter strategy based on FC and *p*-value cut-offs to assess the distributional properties of features in ECM blank and biological samples. The approach makes use of blank samples not incorporated in conventional filtering pipelines, which rely on generic cut-offs (e.g., remove the lowest 40% based on mean/median abundance [43]) and presumably eliminate features of biological relevance. The retention of fewer, but biological relevant features will improve the results of subsequent statistical analysis and facilitate the interpretation of biomarker discovery and drug response phenotyping experiments.

### 2.3. Proof-of-Concept: Early Metabolic Response of CRC Organoids to 5-Fluorouracil Treatment

To proof the feasibility of the optimized protocol C (Supplementary Figure S7) together with the established filtering procedure we investigated the early metabolic response of CRC organoids to 5-fluorouracil (5-FU) treatment. The antimetabolite 5-FU, commonly used in the treatment of colorectal cancer, exerts its anticancer activity through inhibition of thymidylate synthase [44,45] and misincorporation of its metabolites into RNA and DNA [45,46]. Concentrations of 1, 10 and 100 µM (that did not affect cell viability and morphology, Supplementary Figure S9), were used in three independent experiments to induce specific metabolic perturbations within 24 h of treatment. To monitor the repeatability of the whole procedure, the resulting data of each experiment were evaluated independently and then compared.

As a result, non-targeted feature extraction yielded 470–2489 compounds per analytical mode. Further analysis of filtered and sum normalized data revealed, depending on the analytical mode, 3–29 features significantly and relevantly altered upon drug exposure (see Supplementary Table S10). In total, 12 features were significantly and relevantly correlated with 5-FU concentrations in at least two of three experiments (see Table 2). Finally, 10 of those features could be assigned according to levels of assignment proposed by the metabolomics standard initiative (MSI) [47] while two features remained unknown (see Table 2). The consistency of results between the three experiments demonstrates good repeatability of the non-targeted workflow.

**Figure 3:**
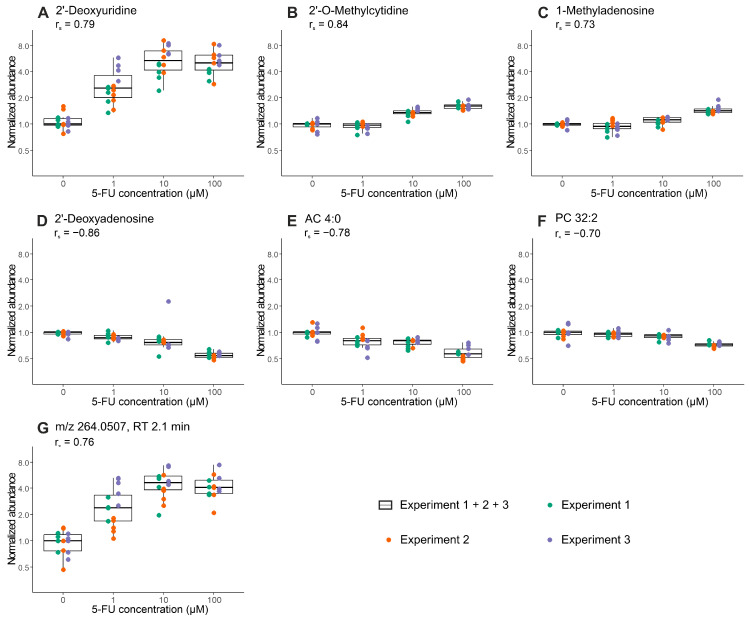
Tukey’s boxplots of dose-dependent changes in metabolite abundance after 24 h of treatment with 5-FU at increasing concentrations assessed in three independent experiments: (**A**) 2′-Deoxyuridine; (**B**) 2′-*O*-Methylcytidine; (**C**) 1-Methyladenosine; (**D**) 2′-Deoxyadenosine; (**E**) AC 4:0; (**F**) PC 32:2; (**G**) feature *m/z* 264.0507 eluting at 2.1 min. Overlaid scatter plots represent individual data points (*n* = 5 technical replicates, except for 100 µM experiment 1 (*n* = 4)) from all three experiments (experiment 1 (green), experiment 2 (orange) and experiment 3 (purple)). Peak areas of individual features were excluded prior to statistical analysis if the measured value was <1% of the group median within the corresponding treatment group (max one value per treatment group). Displayed are all features significantly and relevantly correlated with 5-FU concentration in the joint analysis of all three experiments (Spearman correlation coefficient r_s_ > |0.7| and Benjamini–Hochberg adjusted *p*-value < 0.05). The preprocessed data was normalized to the mean of the corresponding control group.

In a joint analysis of all three experiments, 2′-deoxyuridine, 2′-O-methylcytidine, 1-methyladenosine, 2′-deoxyadenosine, acylcarnitine (AC) 4:0 and phosphatidylcholine (PC) 32:2, and the unassigned feature *m/z* 264.0507 eluting at 2.1 min, still met the applied criteria (Spearman correlation coefficient r_s_ > |0.7| and Benjamini–Hochberg adjusted *p* value < 0.05) for the significant and relevant dose-dependent regulation (see Figure 3).

Most metabolites found to be regulated upon 5-FU treatment are directly involved in pyrimidine and purine metabolism. Our observation of elevated 2′-deoxyuridine and depletion of 2′-deoxyadenosine are largely in accordance with the cellular mechanisms of 5-FU and previous findings in cell culture models [48,49,50,51], rodent derived plasma [48] and clinical trials [52,53]. The observed dose depended increase of inosine levels might be explained by an upregulation of inosine synthesis triggered by increased inosine consumption due to its role as Rib-1-P donor in the activation pathway of 5-FU [54]. The methylated nucleosides 2′-*O*-methylcytidine and 1-methyladenosine occur in different RNA species and are found to be elevated in our experiments. In line with the results presented here, a recent publication describes a considerable increase in the intracellular 1-methyladenosine level after treatment of HCT116 colon cancer cells with 5-FU [55]. In addition tRNA modification by incorporation of 2′-*O*-methylcytidine were previously described in 5-FU-treated *Escherichia coli* [56].

Furthermore, we found an impact on lipid metabolism with decreased levels of AC 4:0, PC 30:0 and PC 32:2. Previous studies in five different CRC cell lines [49] already described an effect of 5-FU treatment on AC metabolism. However, results were not consistent between the different cell lines tested and to some extent in contrast to our findings. In addition, previous studies have reported that increased amounts of phospholipids and altered phospholipid composition of the cell membrane are characteristics of CRC [57,58,59]. Corresponding to this, targeting cancer cells by anticancer treatment could result in decreased PC levels. However, an in-depth biological interpretation of the perturbation of lipid metabolism in 5-FU treated CRC organoids is beyond the scope of this study. We note that results from the proof-of-concept experiment are preliminary and more investigations, carried out in larger cohorts with organoids from different donors, are needed to confirm these findings.

## 3. Materials and Methods

### 3.1. Chemicals and Reagents

Ultra LC-MS grade acetonitrile (ACN) and methanol (MeOH) were purchased from Carl Roth GmbH and Co KG (Karlsruhe, Germany). LC-MS grade methyl tert-butyl ether (MTBE), 2-propanol (IPA), formic acid (FA), ammonium acetate (AmAc) and 5-fluorouracil (5-FU) were purchased from Sigma-Aldrich (Taufkirchen, Germany). Pure water was in-house produced by a Milli-Q system (Millipore, Billerica, MA, USA) and used for the preparation of aqueous solvents. For further details see Supplementary Table S11.

### 3.2. Patient Samples

Colorectal cancer samples were obtained from patients who underwent surgery at the Robert-Bosch-Krankenhaus, Stuttgart. The study was approved by the Ethical Committee at the Eberhard Karls University Tübingen and written informed consent was obtained (project-numbers: 264/2013BO2 and 696/2016BO2). Residual tissue samples not used for pathological routine examination were transferred to the laboratory for cell isolation within a maximum of 8 h after surgery.

### 3.3. Organoid Culture and Viability Assay

Organoid cultures were established and maintained as described previously [4]. Human tumor organoids were cultured in the complete medium (advanced DMEM/F12 (Fisher Scientific/gibco, Grand Island, NY, USA) supplemented with 10 mM Hepes (Carl Roth GmbH and Co KG, Karlsruhe, Germany), 1× Glutamax (Fisher Scientific/gibco, Grand Island, NY, USA), 1× penicillin/streptomycin (Fisher Scientific/gibco, Grand Island, NY, USA), 1× B-27 supplement (Fisher Scientific/gibco, Grand Island, NY, USA), 1× N-2 supplement (Fisher Scientific/gibco, Grand Island, NY, USA), 1 mM *N*-acetylcysteine (Sigma, St. Louis, MO, USA), 50 ng/mL human EGF (Peprotech, London, UK), 10 µM Y-27632 (Absource Diagnostics, München, Germany) and 1.25 µg/mL amphotericin (MERCK, Darmstadt, Germany)).

For cell metabolomics and viability analysis, organoids were dissociated to single cells using the TrypLE Express enzyme (Fisher Scientific/gibco, Paisley, UK). Disaggregation was stopped with advanced DMEM/F12 and cells were counted. Cells were suspended in growth factor-reduced Matrigel^TM^ (Corning, Bedford, MA, USA) and the complete culture medium (3:1, *v/v*). For the protocol evaluation experiments cells were cultured for 3 days in 300 µL of the complete medium prior to analysis. For the proof-of-concept experiments and the viability analysis, a 5-FU stock solution (10 mM 5-FU in water) was diluted with complete medium to final concentrations of 1, 10 and 100 µM 5-FU. After preculturing of the cells for 3 days in 300 µL of complete medium, the medium was replaced by 300 µL of the corresponding 5-FU solution or by the complete culture medium for control (0 µM 5-FU). The organoids were treated for 24 h and then subjected to the metabolomics analysis. The proof-of-concept experiments were performed in 3 independent biological replicates (passage number 39-72).

The CellTiter Glo 3D cell viability assay (Promega, Madison, WI, USA) was used to analyze cell viability according to the manufacturer’s instructions. In brief, an equal volume of reagent was added to the culture medium, mixed thoroughly, incubated for 30 min at room temperature, and transferred into opaque-walled 96-well plates. The intensity of luminescence was measured using the EnSpire plate reader (PerkinElmer, Hamburg, Germany). In addition, cell death was analyzed using the NucRed™ Dead 647 ReadyProbes™ Reagent (ThermoFisher Scientific, Eugene, OR, USA). The reagent was added to the culture medium, incubated for 15 min, and brightfield and fluorescence images (excitation: 642 nm, emission: 661 nm, Cy5 filter cube) were acquired from each well of the 48-well plate using a Cytation 1 Cell Imaging Multi-Mode Reader (BioTek, Winooski, VT, USA). For details about the used reagents and supplements, see Supplementary Table S11.

### 3.4. Sampling and Extraction Procedures

Three protocol variants were compared to find an optimized procedure. The extraction was mainly based on a 2D-cell metabolomics protocol reported by Ivanisevic et al. [18] modified as described below. Each protocol was performed on five organoid sample replicates and three ECM blanks (extracellular matrix without organoids). The samples were collected at room temperature to avoid premature ECM liquefaction. Sample extraction and analysis was performed in a randomized manner.

Protocol A: After removal of the culture medium, 1000 µL of cold PBS (4 °C) was added to each well. Liquefied ECM and organoids were carefully resuspended and transferred into a BSA-coated polypropylene tube (ThermoFisher Scientific, Rockford, IL, USA) sitting on ice. For transfer BSA-coated pipette tips were used. Samples were centrifuged (30 s, 2370× *g*, 4 °C) and the supernatant (PBS-ECM-suspension) was removed. The organoid pellet was resuspended in 100 µL of MeOH/ACN/H_2_O (2:2:1, *v/v/v*) by vortex mixing for approximately 5 s followed by snap-freezing in liquid nitrogen. Samples were stored in liquid nitrogen until further processing.

The samples were thawed at 4 °C and cells were extracted by ultrasonication (ultrasonic wave output power: 320 W, ultrasonic on/off cycles: 0.5 min, total disruption time: 4 min and T = 4 °C; Bioruptor^®^ UCD-200, Diagenode s.a., Liège, Belgium). In order to precipitate proteins, the samples were incubated for at least 30 min at −20 °C, followed by 5 min centrifugation at 21,130× *g* and 4 °C. The resulting supernatant was removed and transferred to a fresh tube. The pellet was resuspended in 100 µL MeOH/ACN/H_2_O (2:2:1, *v/v/v*) on a vortex mixer (approximately 5 s) and centrifugation was repeated. The supernatants were combined and divided in equal aliquots used for HILIC and RPLC analysis.

Protocol B: The samples were extracted as described in protocol A. The remaining organoid pellet was resuspended in 100 µL of MTBE/MeOH (3:1, *v/v*) on a vortex mixer (approximately 5 s) and re-extracted by ultrasonication using the same parameters as before. After centrifugation at 21,130× *g* and 4 °C the supernatant was combined with the aliquot intended for RPLC analysis.

Protocol C: After removal of the culture medium, the surface of the ECM and of the well were washed with 500 µL of warm PBS (37 °C), which was immediately removed and discarded. Cells and ECM were resuspended in 500 µL of ice-cold MeOH/ACN/H_2_O (2:2:1, *v/v/v*), transferred to a polypropylene tube (Eppendorf, Hamburg, Germany) and immediately snap-frozen in liquid nitrogen. Further sample extraction was performed as described for protocol A.

### 3.5. Sample Storage and Preparation

All extract aliquots were evaporated to dryness in a rotational vacuum concentrator (RVC 2-25 CDplus, Christ, Germany) at ambient temperature and stored at −20 °C until analysis. Prior to analysis the dry extracts were reconstituted in 70 μL of solvent (HILIC analysis: ACN:H_2_O (95:5, *v/v*), RPLC analysis: IPA:MeOH (3:1, *v/v*)) by vortex mixing (10 min) and ultrasonication (2 min) followed by centrifugation (5 min, 21,130× *g*, 4 °C). Fifty microliters of the supernatants were transferred into 250 μL glass inserts with polymer feet in 2 mL sample vials (Agilent Technologies, Waldbronn, Germany) covered with preslit polytetrafluoroethylene (PTFE)/silicone screw caps (Agilent Technologies, Waldbronn, Germany). The remaining extracts of all samples (approximately 20 µL each) including ECM blanks were pooled to prepare quality control (QC) samples. Optionally, a 50 µL aliquot was taken for the acquisition of fragmentation spectra while the remaining solution was diluted with the corresponding solvent to achieve an appropriate QC sample volume for monitoring and correction of experimental drifts.

### 3.6. LC-QTOF-MS Analysis

LC/MS analysis was carried out similar as described [17,60]. In brief, aqueous extracts were analyzed by HILIC (Acquity UPLC BEH Amide Column, 1.7 μm, 2.1 mm × 150 mm; Waters, Eschborn, Germany) and organic extracts were analyzed by RPLC (Acquity UPLC BEH C8, 1.7 μm, 2.1 mm × 100 mm; Waters, Eschborn, Germany). Gradient elution at analytical flow rates for HILIC (0.4 mL/min) and RPLC (0.45 mL/min) analysis, each with a total run time of 30 min per sample, was applied (HILIC mobile phase A: 5 mM AmAc and 0.06% FA in water:ACN 1:1, *v/v*, mobile phase B: 5 mM AmAc and 0.06% FA in water:ACN 5:95, *v/v*; RPLC mobile phase A: 5 mM AmAc in water:MeOH 8:2, *v/v*, mobile phase B: 5 mM AmAc in MeOH:ACN:IPA 7.5:2:0.5, *v/v/v*). For both separation systems the autosampler was operated at 6 °C and the column oven at 60 °C. Sample sequence and injection volumes were adjusted as described in Supplementary Table S1. Data acquisition was done using the Mass Hunter Data Acquisition Software (version B.08.00, Agilent Technologies). Fragment spectra were acquired using auto MS/MS analysis in pooled QC samples. Electrospray parameters for MS1 and MS/MS acquisitions were applied as described [17,60]. To obtain the mass accuracy during the batch QTOF reference mass correction (recalibration) was applied according to Leuthold et al. [61].

The calculated amount of <500 cells/injection (see Figure 1) was estimated for the optimized protocol C (Supplementary Figure S7) based on the number of seeded cells (1000 cells/well as determined using a hemocytometer) and a doubling time of 3.4 days. The resulting 2000–3000 cells/well after three days of incubation were subjected to metabolite extraction followed by dividing the extract in equal volumes (one extract for HILIC and one extract for RPLC analysis, see Supplementary Figure S7) resulting in 1000–1500 cells per extract. The dried extracts were reconstituted in 70 µL of solvent from which 20 µL were injected into the LC-MS system thus ending up with an estimated amount of 286–429 cells on column.

### 3.7. Data Preprocessing and Statistical Analysis

Preprocessing of data derived from the non-targeted approach was carried out by using the Mass Hunter Profinder Software (version B.08.00, Agilent Technologies).

#### 3.7.1. Feature Extraction

For protocol assessment, Batch Targeted Feature Extraction on the basis of structurally assigned metabolites [17] was used. Values were matched based on sum formula searching results to mass and retention time with a retention time window match tolerance set to ± 0.7 min and a mass match tolerance set to ± 15 ppm. H^+^, Na^+^ and NH4^+^ adducts were considered for spectra acquired in the positive mode while for negative mode data acquisition the deprotonated molecular ions and CH3COO^−^ and HCOO^−^ adducts were expected. An intensity threshold was not set and an extracted ion chromatogram (EIC) range of ±35 ppm was applied. TOF-MS spectra above 30% of saturation were excluded.

For subsequent experiments non-targeted feature extraction by batch recursive feature extraction (RFE) was applied. The intensity threshold was set to 500–750 counts. Unless specifically stated otherwise, H^+^, Na^+^ and NH4^+^ adducts were selected for positive mode data while the deprotonated molecular ions and CH_3_COO^−^ and HCOO^−^ adducts were expected for negative mode data. The retention time window was set to ±0.2 min, the mass window was set to ± (20 ppm + 2 mDa) and the extracted ion chromatogram (EIC) range to ±35 ppm. For peak integration, Agile2 algorithm was selected. TOF-MS spectra were excluded if their intensity was above 30% saturation. The list of extracted features was inspected visually in order to ensure correct retention time alignment and peak integration throughout the batch. More precisely, extracted ion chromatograms (EICs) of individual features and their chromatographic alignment throughout the analytical batch were reviewed using a graphical interface. Curations were made by manual reintegration of EICs that were falsely integrated by the software algorithm (e.g., correct peak integrations of closely coeluting isomer compounds in two different samples). Such a kind of data curation turned out to be important as poor peak integration and false positive peak detection remains a prevalent problem in untargeted metabolomics data generated using LC-MS [62].

#### 3.7.2. Data Filtration, Normalization and Analysis

Extracted feature data were exported as comma separated value files to perform further data preprocessing and statistical analysis with R-4.0.0 and R studio (http://www.r-project.org) [63], including additional packages (ggplot2 [64], ggrepel [65], ggpubr [66], tidyverse [67], matrixStats [68], matrixTests [69], HybridMTest [70] and ggVennDiagram [71]).

For protocol optimization experiments metabolites with significant and relevant abundance, compared to the metabolite background of the corresponding ECM blank samples, were identified by Welch’s *t*-test on log2 transformed data (*p* < 0.05) and median fold change > 1. In analogy to the commonly applied thresholds used in gene expression analysis [72] the significance level is denoted as 5% throughout the manuscript. The threshold for “relevant” signal intensities is specified by the indicated fold-change thresholds of the different experiments.

In the proof-of-concept experiments, signal drifts were corrected using locally weighted scatterplot smoothing (LOESS) correction over QC samples. Features with a coefficient of variation (CV) ≥20% in QC samples analyzed throughout the batch were removed from the data after LOESS. Furthermore, data was filtered for features with significant and relevant abundance compared to the mean of the corresponding ECM blank samples. Therefore, Welch’s *t*-test was applied to log2 transformed data of the control and ECM blank samples. Significant and relevant features with a fold-change of >1.2 were included in further data analysis. A signal intensity variability of <20% is well established to remove features of low reproducibility in non-targeted metabolomics [32,73,74]. Based on this assumption, that a variability of <20% is acceptable and therefore non-relevant, we conversely concluded that a difference of >20% (e.g., a > 1.2-fold abundance of a feature in organoids compared to ECM blanks) is of relevance.

Filtered data was normalized (peak area of each feature divided by the sum of peak areas of all features in one sample). Significantly and relevantly dose-dependent regulated features were identified by Spearman correlation analysis, considering r_s_ > |0.7| and Benjamini-Hochberg adjusted [75] *p*-value < 0.05. All statistical tests were two sided.

### 3.8. Metabolite Identification and Annotation

Metabolite identification and annotation was performed based on accurate mass and RT-matching for reported compounds from targeted feature extraction whereas two of the following criteria had to be met: mass tolerance: ± 15 ppm, retention time tolerance: ± 0.2 min and a targeted matching score >70% with the following weightings for score calculation: mass score: 100%; isotope abundance score: 60%; isotope spacing score: 50% and retention time score: 20%.

In the proof-of-concept experiments metabolite identification and annotation was performed by comparison of spectral information (accurate mass, fragment ions and/or retention time) acquired in QC samples to available spectral information from databases or from pure standard compounds. MS/MS spectra were accessed by the Mass Hunter Qualitative Analysis Software (Version B.07.00, Agilent Technologies) and spectral matching was assessed based on scores reported by the indicated search engine (Supplementary Tables S2–S5): MassBank of North America (MS/MS Similarity Search, https://mona.fiehnlab.ucdavis.edu/), CEU Mass Mediator (MS/MS Search) [39,40] and Lipid Annotator (Version 1.0, Agilent Technologies). Score values for spectra matching to selected reference compounds or those provided in the METLIN Metabolite PCDL (Version B.07.00, Agilent Technologies) were obtained by spectral comparison within the MassHunter PCDL Manager Software (Version B.07.00, Agilent Technologies). Assignment levels proposed by the Metabolomics Standard Initiative (MSI) [47] are provided in the Supplementary Tables S2–S5.

## 4. Conclusions

To the best of our knowledge this is the first study on method optimization for non-targeted metabolic and lipidomic profiling of ECM-based organoid cultures. We could show that reliable and repeatable data acquisition of a broad metabolic range is possible from the extract of less than 500 cells per injection via untargeted LC-QTOF-MS. This rapid and sensitive procedure enables the determination of the early metabolic response of CRC organoids to 5-FU treatment and paves the way for high throughput investigations of metabolic changes in patient derived CRC organoids.

In future projects, an adaption of the new protocol for metabolic flux profiling in 3D-organoid models by non-targeted stable isotope labeling analysis [76] may improve the understanding of pathobiochemical mechanisms and drug response effects. In this regard, the implementation of non-targeted isotope dilution normalization [77] may facilitate the quantification of unidentified features in a retrospective fashion.

## Figures and Tables

**Table 1:** Number of metabolites with significantly and relevantly higher abundance in organoid samples compared to respective controls (ECM only) following different sample preparation protocols and LC QTOF-MS methods in the indicated electrospray ionization (ESI) mode.

Protocol	Analytical Mode	No. of Significant and Relevant Metabolites (Organoids vs. ECM Controls)	Median CV of Significant and Relevant Metabolites (%)
A	RPLC ESI (−)	17	21.7
	RPLC ESI (+)	12	14.7
B	RPLC ESI (−)	13	7.0
	RPLC ESI (+)	13	8.9
A/B ^1^	HILIC ESI (−)	15	25.7
	HILIC ESI (+)	19	33.5
C	RPLC ESI (−)	44	13.6
	RPLC ESI (+)	54	10.4
	HILIC ESI (−)	17	26.8
	HILIC ESI (+)	25	16.2

^1^ Data of protocols A and B was combined for statistical evaluation, as sample preparation for both protocols is identical in the HILIC mode, see Figure 1.

**Table 2:** Features significantly and relevantly altered upon 5-FU treatment of CRC organoids.

Analytical Mode	No. of Experiments ^1^	Mean Mass	Retention Time	Regulation	Annotation	MSI Level ^4^
HILIC ESI (+)	3	111.0436	3.21	↑	Cytosine ^2^	2
		251.1026	2.42	↓	2′-Deoxyadenosine	1
		257.1022	3.21	↑	2′-O-Methylcytidine	1
	2	231.1468	5.95	↓	AC 4:0	2
		268.0828	4.89	↑	Inosine	2
		281.1115	7.90	↑	1-Methyladenosine	1
		633.4739	3.78	↓	LysoPC 26:1	2
HILIC ESI (−)	3	228.0731	2.12	↑	2′-Deoxyuridine	2
		264.0507	2.12	↑	na ^3^	-
	2	536.1892	2.17	↑	na	-
RPLC ESI (+)	2	705.5341	6.75	↓	PC 30:0	2
		729.5347	6.48	↓	PC 32:2	2

^1^ No. of experiments where the applied criteria for significant and relevant response to 5-FU treatment are met. ^2^ In-source fragment of 2′-*O*-methylcytidine. ^3^ Supposed to be related to uracil due to detection of m/z 111.0211 in the fragment spectra of 264.0507. ^4^ Assignment level according to the metabolomics standard initiative (MSI) [47]. AC, acylcarnitine; LysoPC, lysophosphatidylcholine; PC, phosphatidylcholine; na, not assigned.

## Supplementary Materials

The following are available online at https://www.mdpi.com/2218-1989/10/12/494/s1. Supplementary Figures: Figure S1: Metabolites found to be of significant and relevant abundance in organoid samples compared to corresponding ECM blank samples (protocols A/B and C in HILIC ESI (+) mode). Figure S2: Metabolites found to be of significant and relevant abundance in organoid samples compared to corresponding ECM blank samples (protocols A/B and C in HILIC ESI (−) mode). Figure S3: Metabolites found to be of significant and relevant abundance in organoid samples compared to corresponding ECM blank samples (protocols A, B and C in RPLC ESI (+)). Figure S4: Metabolites found to be of significant and relevant abundance in organoid samples compared to corresponding ECM blank samples (protocols A, B and C in the RPLC ESI (−) mode). Figure S5: Venn diagrams displaying the overlap of the tested extraction procedures with respect to metabolites present in organoid samples with significant and relevant abundance. Figure S6: Venn diagrams displaying the extent of overlap between the different analytical modes for significantly and relevantly detected metabolites in protocol C. Figure S7: Optimized protocol for comprehensive and reproducible metabolomic and lipidomic profiling of CRC organoids using LC-QTOF-MS after dual LC separation. Figure S8: Influence of the established data filtering procedure on data quality with regard to the variability of retained features in the HILIC ESI (+) mode, HILIC ESI (−) mode, RPLC ESI (+) mode and RPLC ESI (−) mode. Figure S9: Exemplary pictures from preliminary experiments to ensure cell viability at the time of sampling. Supplementary Table S1: Analytical batch structure. Table S2: Putatively annotated/identified compounds in the HILIC ESI (+) mode. Table S3: Putatively annotated/identified compounds in the HILIC ESI (−) mode. Table S4: Putatively annotated/identified compounds in the RPLC ESI (+) mode. Table S5: Putatively annotated/identified compounds in the RPLC ESI (−) mode. Table S6: Search result from the accurate mass batch search (CEU mass mediator) of compounds found with higher abundance in ECM blank samples—HILIC ESI (+) mode. Table S7: Search result from the accurate mass batch search (CEU mass mediator) of compounds found with higher abundance in ECM blank samples—HILIC ESI (−) mode. Table S8: Search result from the accurate mass batch search (CEU mass mediator) of compounds found with higher abundance in ECM blank samples—RPLC ESI (+) mode. Table S9: Search result from the accurate mass batch search (CEU mass mediator) of compounds found with higher abundance in ECM blank samples—RPLC ESI (−) mode. Table S10: Significantly and relevantly regulated features detected upon 5-flurouracil treatment—all modes. Table S11: Used chemicals and reagents. Metabolomics data have been deposited to the EMBL-EBI MetaboLights database [78] with the identifier MTBLS2130.
