# Supplementary material for: Metabolic Drug Response Phenotyping in Colorectal Cancer Organoids by LC-QTOF-MS
Source: Metabolites. 2020 Dec 1;10(12):494. doi: 10.3390/metabo10120494 (PMC7760698; doi:10.3390/metabo10120494)
Supplement: Supplementary file 1 [file metabolites-10-00494-s001.zip › Neef_et_al_Supplementary_Figures_final.pdf]

Supplementary Figures

Metabolic Drug Response Phenotyping in Colorectal Cancer Organoids by LC-QTOF-MS

Sylvia K. Neef [1,#], Nicole Janssen [1,#], Stefan Winter [1], Svenja K. Wallisch [1], Ute Hofmann [1], Marc H. Dahlke [1,2], Matthias Schwab [1,3,4], Thomas E. Mürdter [1,#] and Mathias Haag [1,#,*]

[1] Dr. Margarete Fischer-Bosch-Institute of Clinical Pharmacology, Stuttgart, Germany and University of Tuebingen, Tuebingen, Germany
[2] Department of Surgery, Robert-Bosch Hospital, Stuttgart, Germany
[3] Departments of Clinical Pharmacology, and of Pharmacy and Biochemistry, University of Tuebingen, Tuebingen, Germany
[4] Cluster of Excellence iFIT (EXC 2180) „Image-Guided and Functionally Instructed Tumor Therapies", University of Tuebingen, Germany
* Correspondence: mathias.haag@ikp-stuttgart.de; Phone +49 (0)711 / 8101-5429
[#] contributed equally

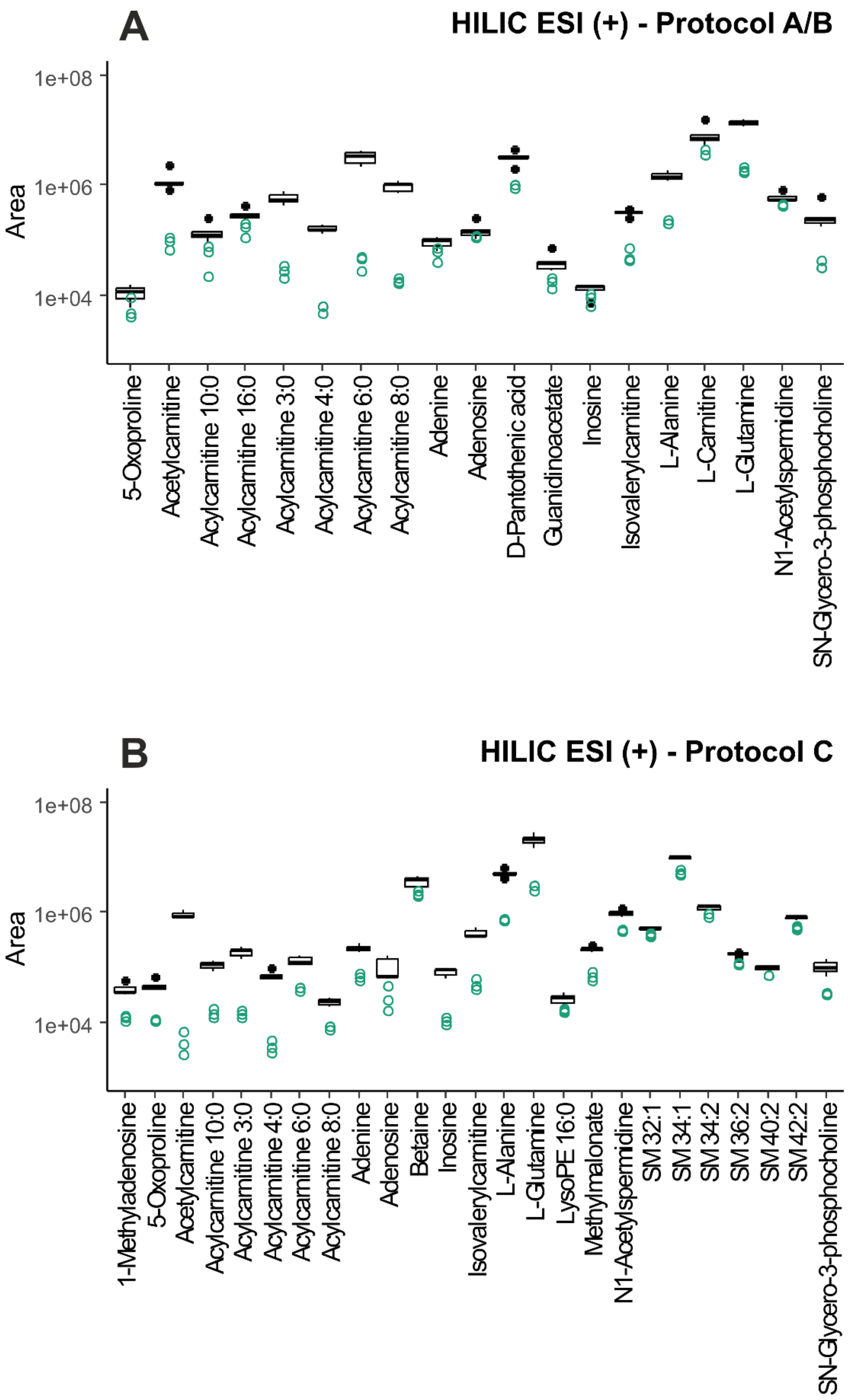

Figure S1. Metabolites found to be of significant and relevant abundance in organoid samples (black boxplots, $n = 10$ technical replicates, protocols A/B; $n = 5$ technical replicates, protocol C) compared to corresponding ECM blank samples (green circles, $n = 3$ technical replicates): **A** HILIC ESI (+) results of protocol A/B (data of these protocols was combined for statistical evaluation since sample preparation is identical for both protocols, see figure 1); **B** HILIC ESI (+) results of protocol C;

LysoPE, lysophosphatidylethanolamine; SM, sphingomyelin.

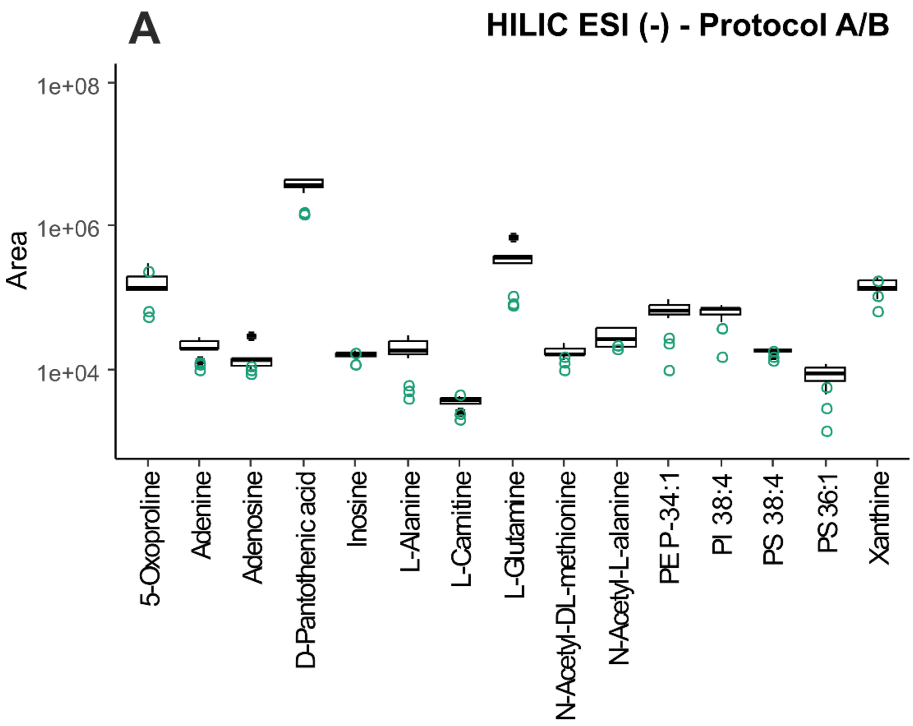

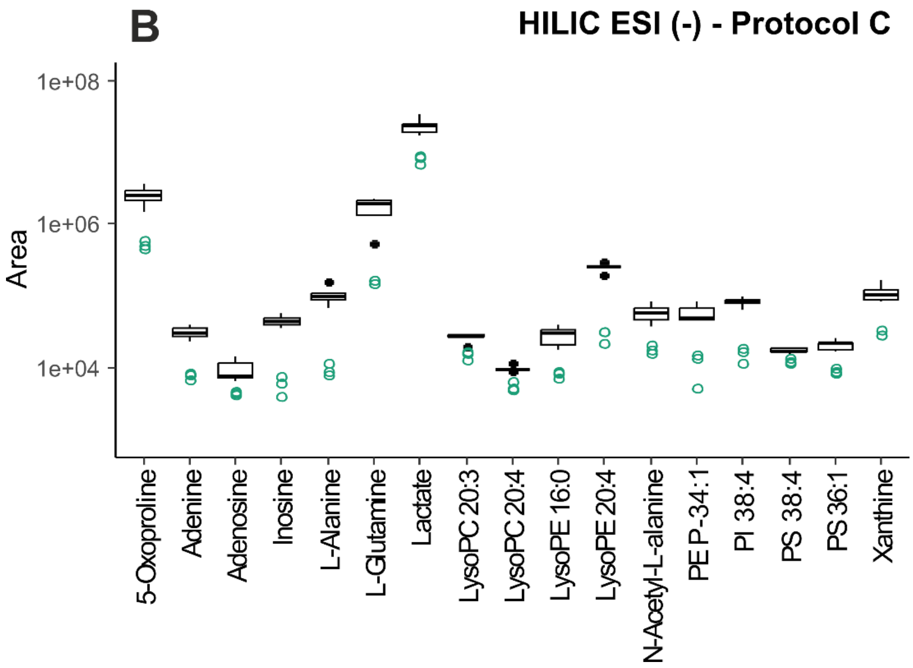

Figure S2. Metabolites found to be of significant and relevant abundance in organoid samples (black boxplots, *n* = 10 technical replicates, protocols A/B; *n* = 5 technical replicates, protocol C) compared to corresponding ECM blank samples (green circles, *n* = 3 technical replicates): **A** HILIC ESI (-) results of protocol A/B (data of these protocols was combined for statistical evaluation since sample preparation is identical for both protocols, see figure 1); **B** HILIC ESI (-) results of protocol C;

LysoPC, lysophosphatidylcholine; LysoPE, lysophosphatidylethanolamine; PE, phosphatidylethanolamine; PI, phosphatidylinositol; PS, phosphatidylserine.

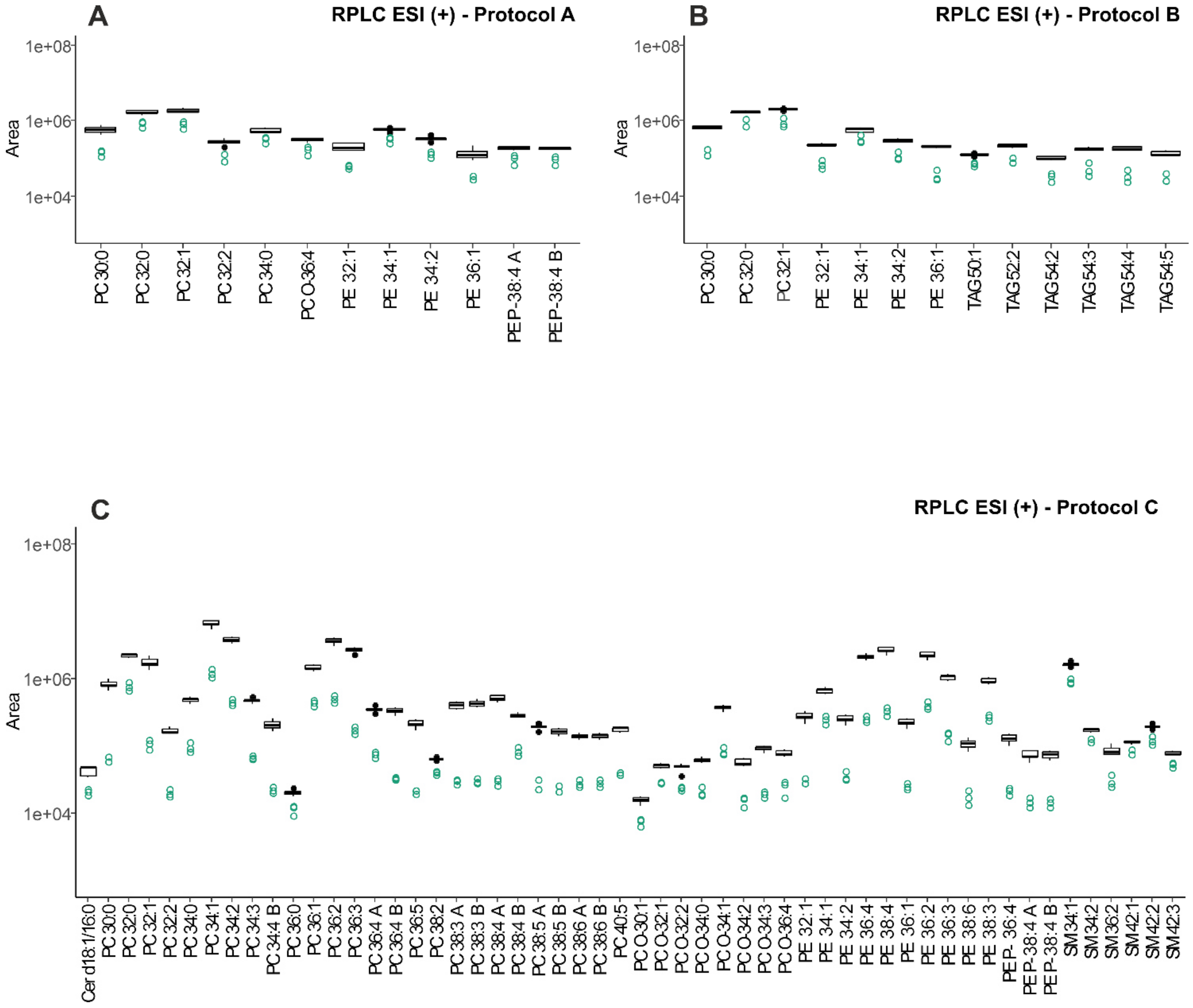

Figure S3. Metabolites found to be of significant and relevant abundance in organoid samples (black boxplots, *n* = 5 technical replicates) compared to corresponding ECM blank samples (green circles, *n* = 3 technical replicates): **A** RPLC ESI (+) results of protocol A; **B** RPLC ESI (+) results of protocol B; **C** RPLC ESI (+) results of protocol C.

Cer, ceramide; PC, phosphatidylcholine; PE, phosphatidylethanolamine; SM, sphingomyelin; TAG, triacylglycerol.

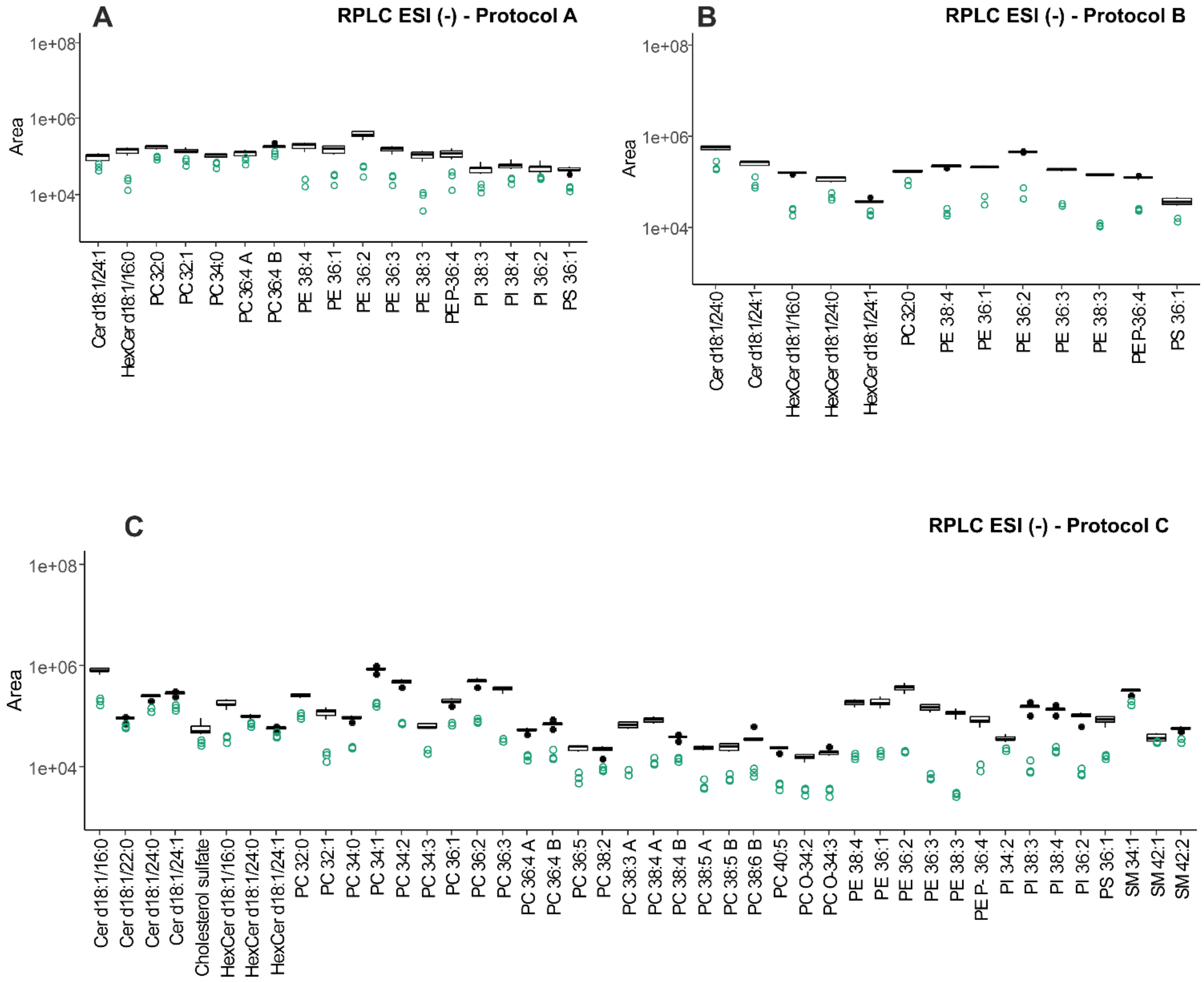

Figure S4. Metabolites found to be of significant and relevant abundance in organoid samples (black boxplots, *n* = 5 technical replicates) compared to corresponding ECM blank samples (green circles, *n* = 3 technical replicates): **A** RPLC ESI (-) results of protocol A; **B** RPLC ESI (-) results of protocol B; **C** RPLC ESI (-) results of protocol C.

Cer, ceramide; HexCer, hexosylceramide; PC, phosphatidylcholine; PE, phosphatidylethanolamine; SM, sphingomyelin; PI, phosphatidylinositol; PS, phosphatidylserine.

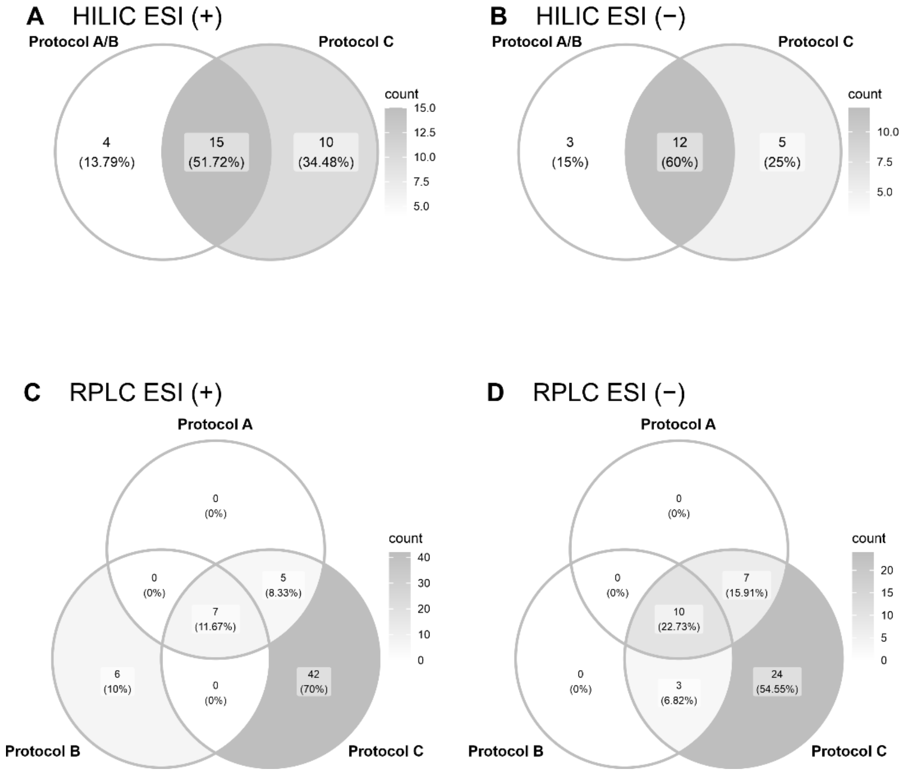

Figure S5. Venn diagrams displaying the overlap of the tested extraction procedures (see Figure 1) with respect to metabolites present in organoid samples with significant and relevant abundance. **A:** HILIC ESI (+) mode, **B:** HILIC ESI (-) mode, **C:** RPLC ESI (+) mode and **D:** RPLC ESI (-) mode. Since protocols A and B are identical for samples analyzed via HILIC, they were evaluated together (diagrams A and B).

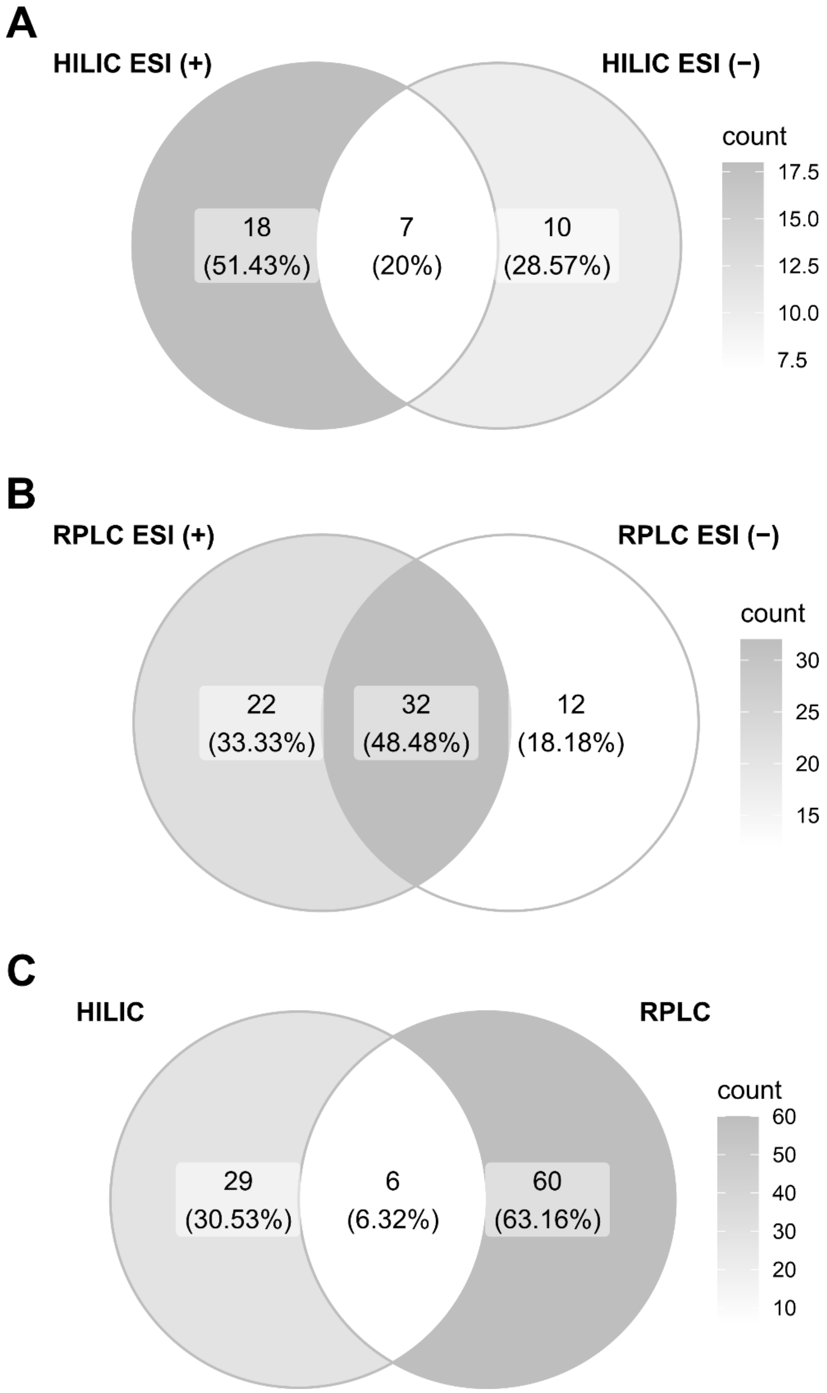

Figure S6. Venn diagrams displaying the extent of overlap between the different analytical modes for significantly and relevantly detected metabolites in protocol C (see Supplementary Figures S1-S4): **A** HILIC ESI(+) and HILIC ESI(-), **B** RPLC ESI(+) and RPLC ESI(-), and **C** HILIC and RPLC.

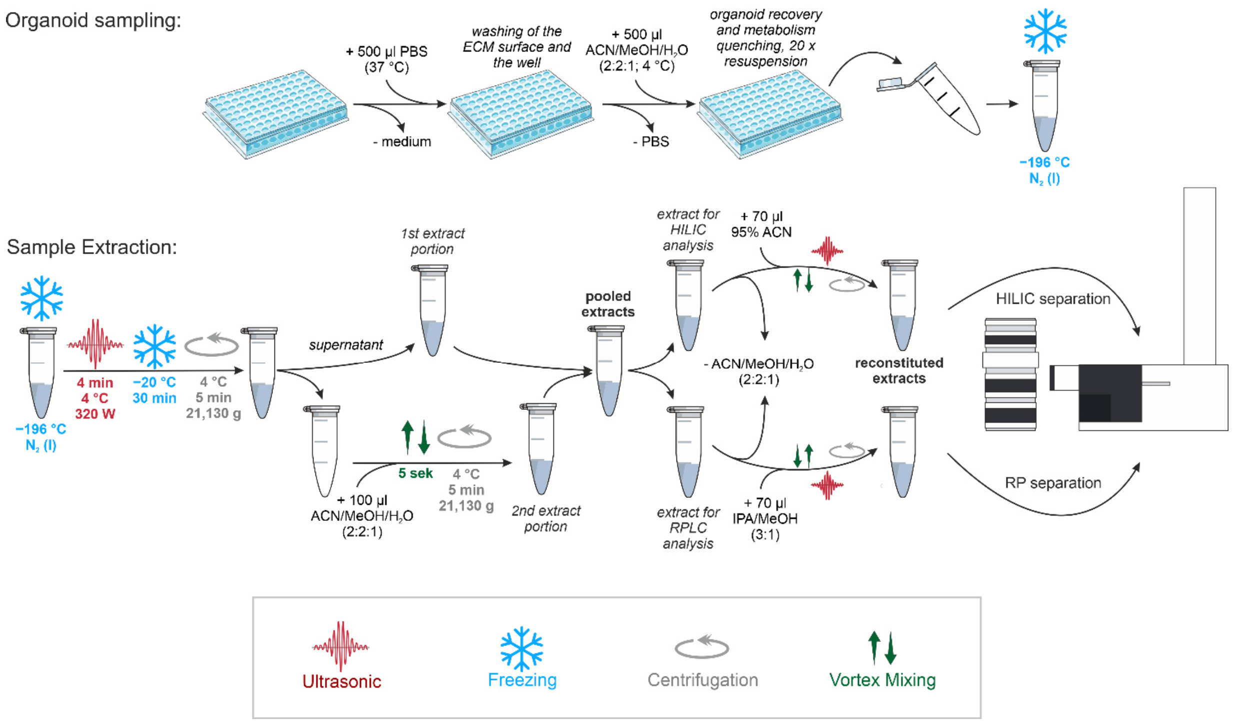

Figure S7. Optimized protocol for comprehensive and reproducible metabolomic and lipidomic profiling of CRC organoids using LC-QTOF-MS after dual LC separation by HILIC and RPLC. The red wave icon indicate ultrasonic cell extraction with on/off cycles of 0.5 min and total disruption time of 4 min. Blue snow flake icons represent sample freezing. Grey and green arrows display centrifugation and vortex mixing, respectively.

PBS, phosphate-buffered saline; ACN, acetonitrile; MeOH, methanol; IPA, isopropanol.

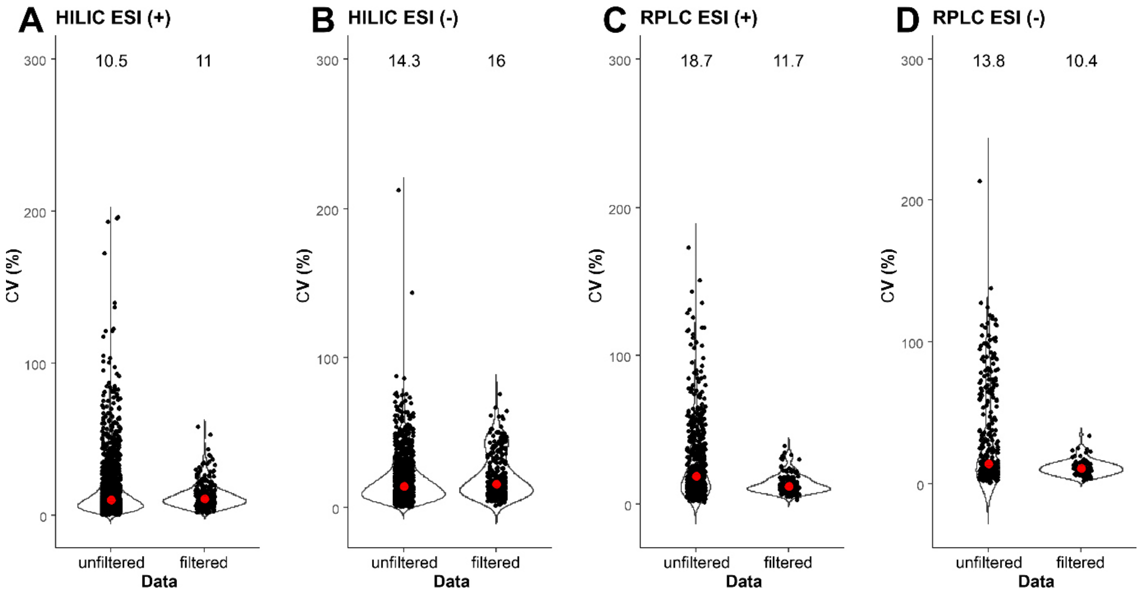

Figure S8. Influence of the established data filtering procedure on data quality with regard to the variability of retained features. **A:** HILIC ESI (+) mode, **B:** HILIC ESI (-) mode, **C:** RPLC ESI (+) mode and **D:** RPLC ESI (-) mode. A fold change (FC) of 1.2 (untreated organoid samples/ECM blank samples) and a significance level of 5% (uncorrected p-value < 0.05) were applied as filter cut-offs. Bean plots representing the coefficients of variation (CVs, $n = 5$ technical replicates) of features (black dots) detected in untreated organoid samples before and after data filtering workflow. Median CVs of each single mode are indicated by red dots and listed above the beanplots.

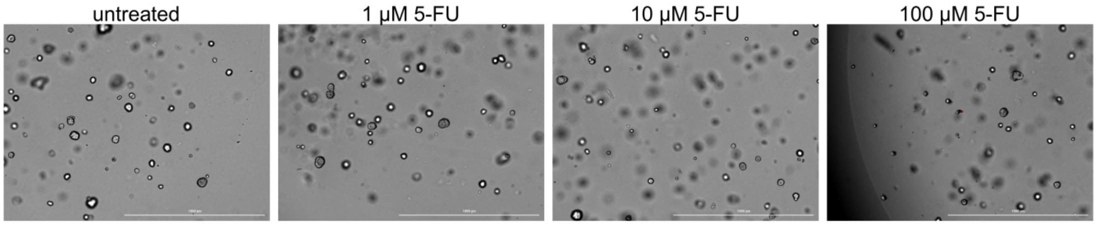

Figure S9. Exemplary pictures from preliminary experiments to ensure cell viability at the time of sampling. The nuclei of dead cells were stained with NucRed™ Dead 647 ReadyProbes™ Reagent (far-red).
